# Infections following medical leech therapy for rhizarthrosis: a case series

**DOI:** 10.1080/23320885.2026.2741520

**Published:** 2026-10-15

**Authors:** Laura Coenen de Salazar, Stefan Weber, Martin Lautenbach

**Affiliations:** a Department of Hand Surgery, upper extremity and foot surgery, Berlin Center for Orthopedic Rheumatology, Waldfriede Hospital, Berlin, Germany; b Department of Trauma, Reconstructive and Rehabilitation Surgery, University Medicine Greifswald, Greifswald, Germany

**Keywords:** Rhizarthrosis, medical leech therapy, infection, thumb carpometacarpal joint empyema

## Abstract

Medical leech therapy is used as an alternative treatment for chronic pain conditions, including rhizarthrosis. During leech therapy, the leech releases a variety of bioactive substances that are intended to alleviate symptoms and relieve pain. The procedure is often described as a ‘natural’ and risk-free form of naturopathic treatment. However, medical leech therapy is associated with risks and potential complications, among which infection is one of the most common. According to the literature, these infections may be caused by Aeromonas spp. as well as other pathogens. Treatment typically consists of intravenous antibiotic therapy combined with surgical intervention. We report a case series of five patients who presented to the Department of Hand Surgery at Waldfriede Hospital with infections following medical leech therapy for rhizarthrosis. No consistent microbiological pattern of causative pathogens was observed. Several strategies for preventing infections associated with medical leech therapy have been described in the literature. Nevertheless, serious infectious complications cannot be entirely excluded.

## Introduction

The medical use of leeches has a long history. The earliest evidence of the medical use of leeches dates back to ancient Egypt [1,2]. Today, medical leech therapy is used in plastic and reconstructive surgery, among other indications, for the treatment of venous congestion following free and pedicled flap procedures or after digital replantation [1,3–5]. However, the procedure is also used for treatment of rhizarthrosis, gonarthrosis, chronic pain, and venous disorders [2,5–7]. For example, Michalsen et al. reported good efficacy of leech therapy in terms of pain relief in rhizarthrosis [6]. In the early stages of rhizarthrosis (Eaton/Littler I and II), the established treatment is generally conservative. This usually involves the use of orthoses and nonsteroidal anti-inflammatory drugs (NSAIDs). Intra-articular injections of corticosteroids and hyaluronic acid, as well as radiosynoviorthesis, physical therapy, and occupational therapy, have also been described in the literature. In advanced stages (Eaton/Littler III and IV) or when conservative treatment fails in the early stages, surgical intervention is indicated. Established surgical treatment options include resection arthroplasty and joint arthroplasty [3].

During medical leech therapy, leeches are applied to the cleansed skin over the affected area. Michalsen et al. even advise against prior skin disinfection, as this may negatively affect the leech’s feeding behavior. Furthermore, shaving heavily haired areas and rubbing the skin until erythema develops may facilitate the feeding process. A leech typically ingests between 5 and 15 mL of blood, and the suction process usually lasts between 20 and 60 min [4,8]. During this process, bioactive substances are released *via* the leech’s saliva (according to the literature, the saliva contains over 20 bioactive substances [7]). Among other things, these include analgesic and anti-inflammatory (including antistasin, hirustasin), anticoagulant (including hirudin, factor Xa inhibitors), vasodilatory (including histamine-like molecules), and platelet-inhibiting substances (including saratin, calin, apyrase, decorsin) [2,4,7].

Leeches are known to live in symbiosis with the bacterium Aeromonas hydrophila. This bacterium is found in the leech’s intestinal tract [1,4].

The most common complications of medical leech therapy include infections, allergies, and prolonged bleeding. Cases of meningitis and microangiopathy have also been reported following leech therapy [2,5]. Verriere et al. reported an infection rate following medical leech therapy of between 2.4% and 36.2% [2]. These infections are most commonly caused by Aeromonas hydophila [1,2,5]. Other pathogens described in the literature include Aeromonas sobria, Aeromonas caviae, Serratia marcescens, and Enterobacter aerogenes [9].

In the treatment of Aeromonas hydrophila infections, ciprofloxacin, sulfamethoxazole/trimethoprim, and second- and third-generation cephalosporins are of particular importance [5]. Nevertheless, resistance of Aeromonas hydrophila strains to ciprofloxacin has been reported in several cases [1,4].

We report a case series of five patients who presented to the Department of Hand Surgery at Waldfriede Hospital with infections following medical leech therapy for rhizarthrosis. The patients gave their verbal consent to participate in the study.

## Patients 1 and 2

In our case report, two patients from the same family (father and son, aged 63 and 34) underwent medical leech therapy. Both patients were right-handed and worked in office-based occupations. Medical leech therapy was performed due to pain in the thumb carpometacarpal joint. The patients reported a history of medical leech therapy performed at six-month intervals over the previous two years. The leeches had been applied to the area of the thumb carpometacarpal joint after the skin had been cleansed. Twenty-four hours after treatment, both patients presented with localized redness and swelling. They also experienced pain. As the local signs of infection progressed, they presented to the Emergency Department of Waldfriede Hospital two days after the leech therapy. At presentation, both patients showed signs of infection (Figures 1–4). Laboratory tests revealed mildly elevated inflammatory parameters (CRP 0.5 mg/dL and leukocytes 9,880/µL, and CRP 0.73 mg/dL and leukocytes 11,300/µL, respectively). Imaging of the 34-year-old patient (son) revealed only mild signs of rhizarthrosis (Eaton/Littler stage I) (Figures 5 and 6). In the 63-year-old patient (father), rhizarthrosis was classified as Eaton/Littler stage II at the time of presentation (Figures 7 and 8). Both patients required emergency surgical intervention on the day of presentation. In both cases, extensive wound debridement, including excision of infected tissue, irrigation, and drain placement was performed. The drains were removed after three days. A second-look surgical revision was not required in either case. Postoperatively, the inflammatory parameters as well as the local redness and swelling subsided. Intraoperative cultures from the 63-year-old patient revealed growth of S. epidermidis. In the case of the 34-year-old patient, no pathogen could be detected. Both patients were treated with cefuroxime at a dose of 1.5 g intravenously three times daily throughout their hospital stay. The antibiogram demonstrated that the S. epidermidis strain was susceptible to cefuroxime. Father and son were discharged after seven days with uncomplicated wound healing. Both patients wore a splint until suture removal after 14 days. At discharge, antibiotic therapy was switched to oral amoxicillin/clavulanic acid. This was continued for an additional 7 days. At the completion of treatment, both patients had a full range of motion in all planes of the affected hand.

**Figures 1 and 2.**
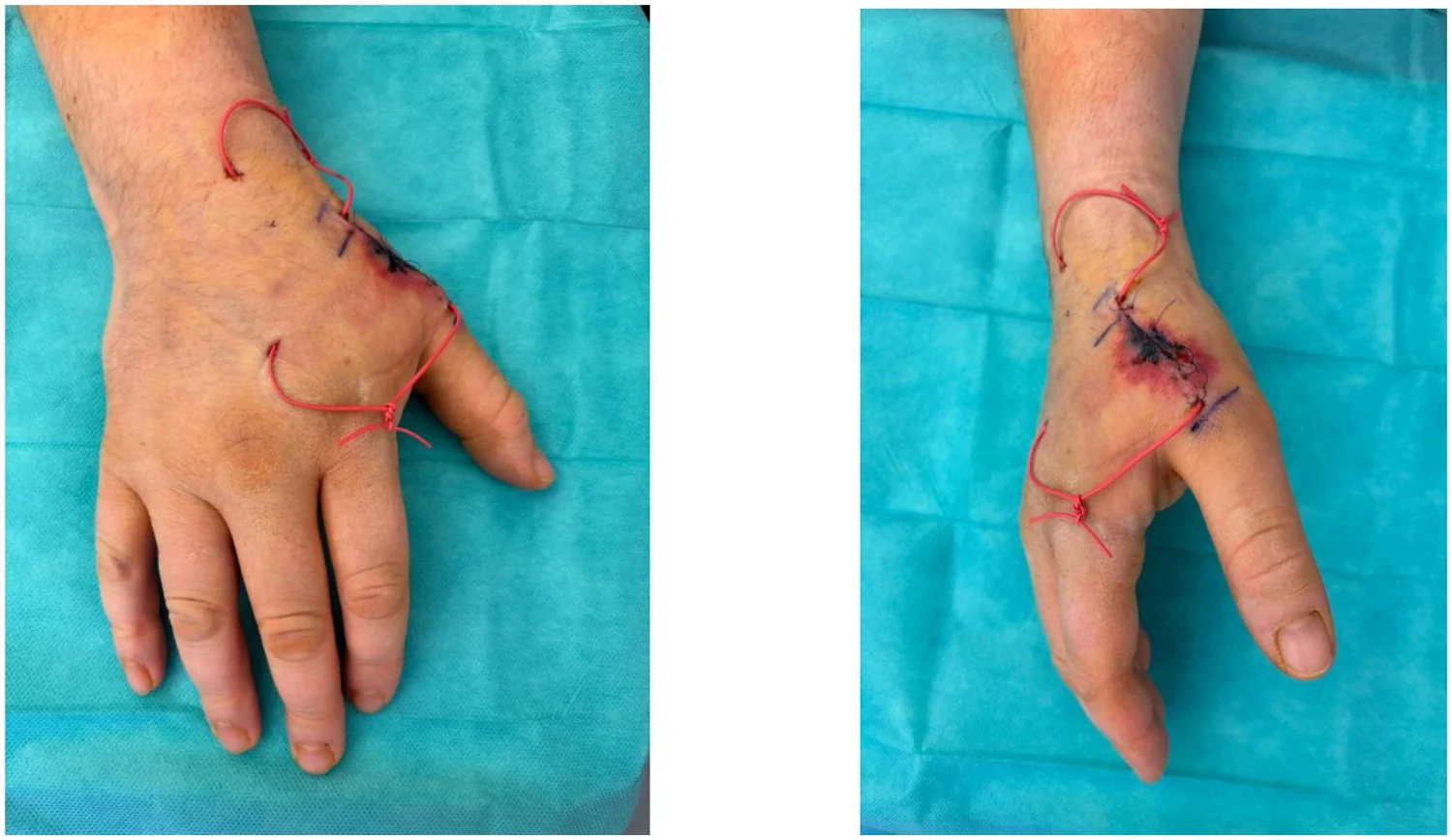
34-year-old patient: postoperative findings following debridement and placement of vessel loops.

**Figures 3 and 4.**
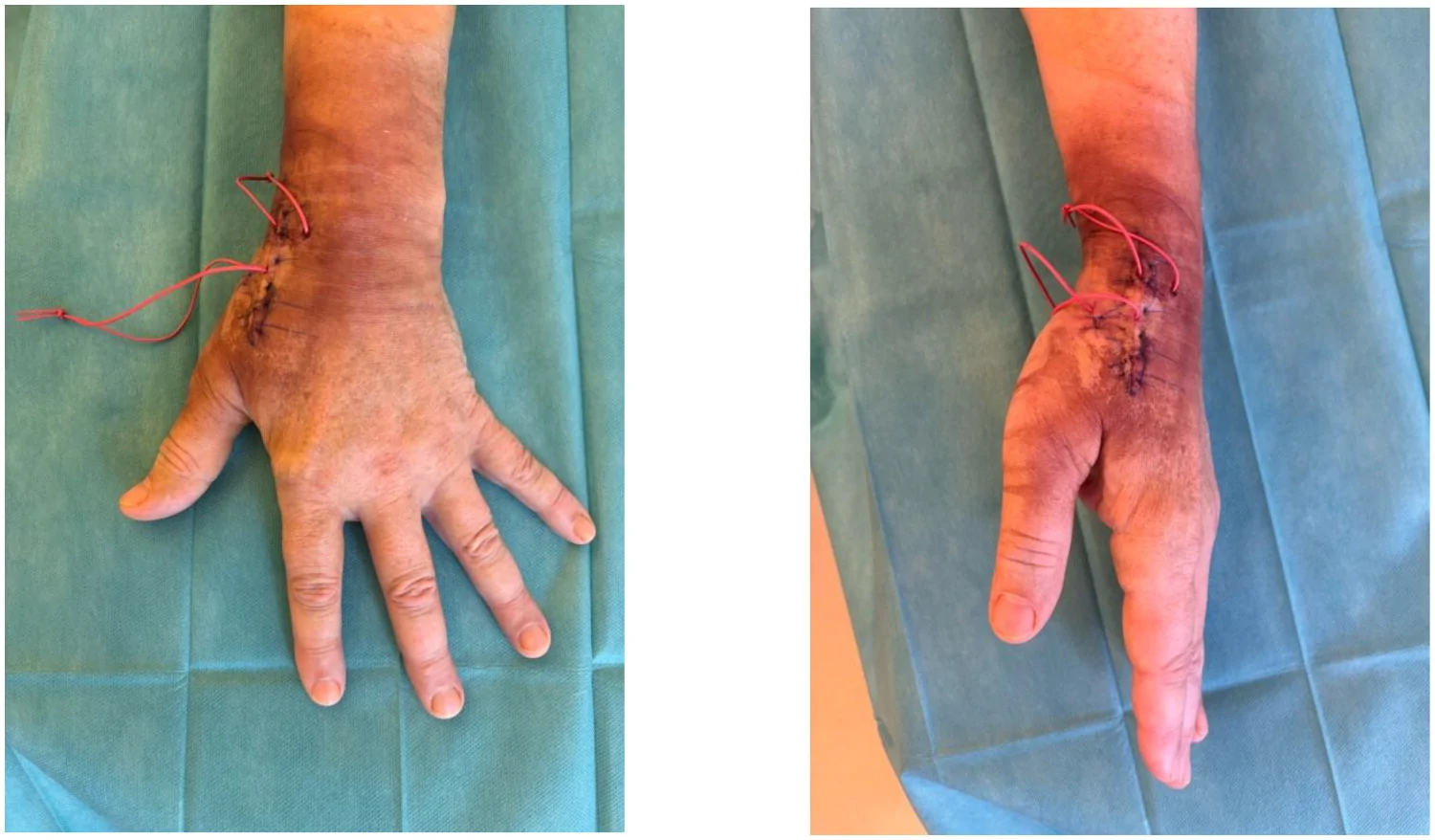
63-year-old patient: postoperative findings following debridement and placement of vessel loops.

**Figures 5 and 6.**
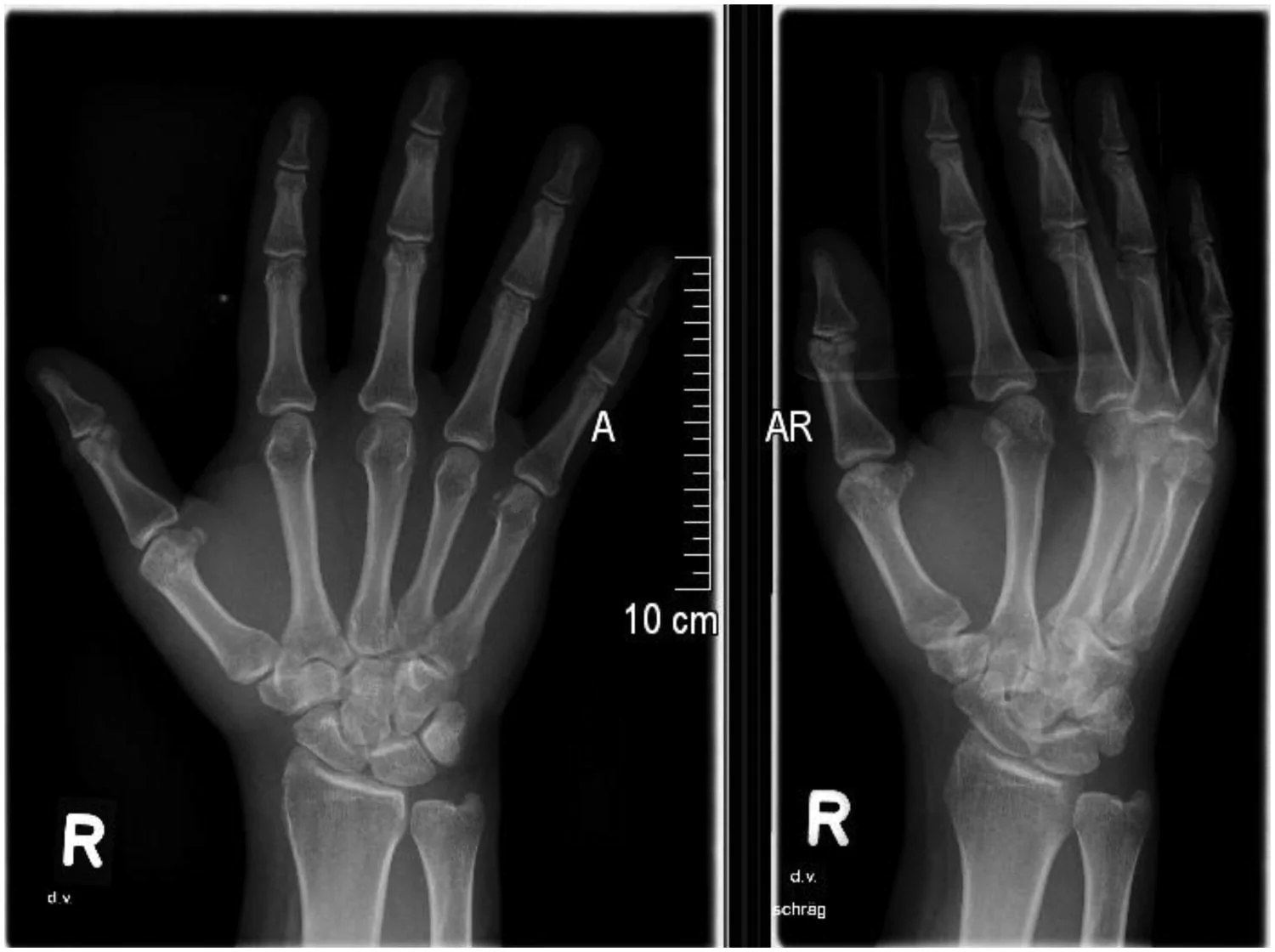
34-year-old patient: radiographs of the right hand at presentation (Eaton/Littler stage I).

**Figures 7 and 8.**
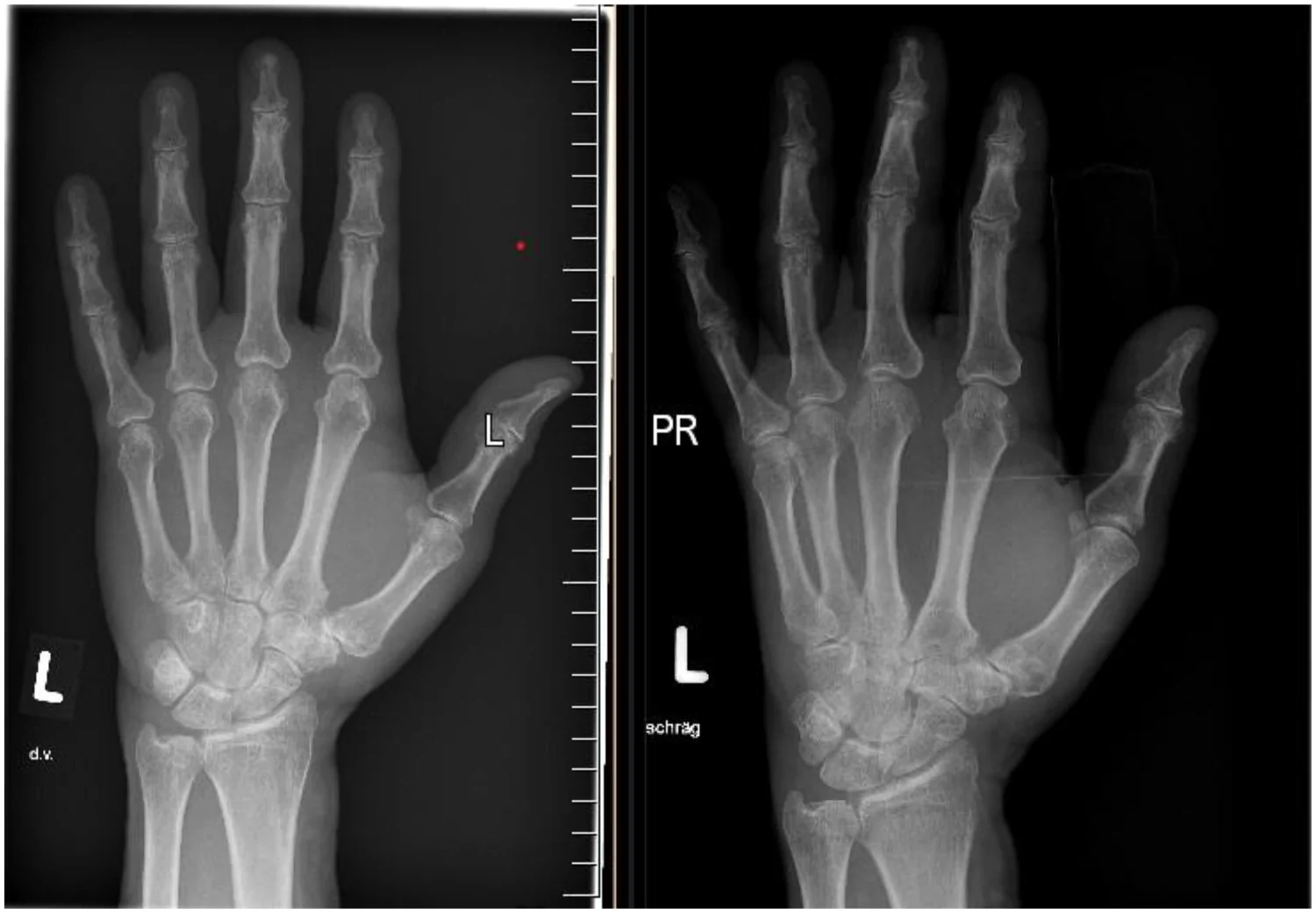
63-year-old patient: radiographs of the left hand at presentation (Eaton/Littler stage II).

## Case 3

A 58-year-old man presented with an infection of the right hand following medical leech therapy. Redness, swelling, and pain developed 48 h after treatment, which prompted presentation to our hospital. At the time of presentation, imaging revealed Eaton/Littler stage II rhizarthrosis. No elevated inflammatory parameters were detected on laboratory testing. The patient underwent surgery on the day of presentation. Intraoperatively, pus was identified within the thumb carpometacarpal joint, consistent with septic arthritis and empyema of the thumb carpometacarpal joint. A second-look procedure was subsequently required. Intraoperative cultures revealed growth of A. hydrophila. During his hospital stay, the patient was treated with intravenous ciprofloxacin. The duration of hospitalization was five days. Splint immobilization was maintained for 14 days. At the completion of treatment, the patient demonstrated a full range of motion of the affected hand in all planes despite progression of rhizarthrosis to Eaton/Littler stage IV.

## Case 4

A 64-year-old woman presented with a localized infection of her right hand following medical leech therapy. The symptoms (redness, swelling, pain, and a throbbing sensation) first appeared 24 h after treatment. Laboratory tests revealed a mildly elevated CRP level of 1.5 mg/dL, while the leukocyte count remained within the normal range. Initially, rhizarthrosis was classified as Eaton/Littler stage III. Intraoperatively, synovitis was present, without evidence of pus. Synovectomy of the abductor pollicis longus (APL), extensor pollicis brevis (EPB), and extensor pollicis longus (EPL) tendons was performed. Microbiological cultures were positive for Pseudomonas aeruginosa. Initially, the patient received intravenous cefuroxime for five days. Following receipt of the microbiological results and the antibiogram, antibiotic therapy was switched to piperacillin/tazobactam according to the susceptibility profile. Intravenous antibiotic therapy was continued for a total of nine days. Two additional surgical procedures were required to control the infection. At the completion of treatment, the patient demonstrated good function of the affected hand. However, the follow-up showed progression of rhizarthrosis to Eaton/Littler stage IV.

## Case 5

Forty-eight hours after undergoing medical leech therapy, a 58-year-old woman presented with an ­infection of her right hand characterized by localized redness, swelling, and pain, with a CRP level of 1.8 mg/dL and a leukocyte count within the normal range. Initial imaging demonstrated Eaton/Littler stage II rhizarthrosis. The infection was treated surgically. Intraoperatively, copious pus was identified extending into the thumb carpometacarpal joint, consistent with septic arthritis and empyema of the thumb carpometacarpal joint. A second-look procedure was required for further surgical management of the infection. Intraoperative cultures revealed growth of Staph. aureus. The patient was subsequently treated with intravenous flucloxacillin for six days. Follow-up demonstrated progression of rhizarthrosis to Eaton/Littler stage III.

Table 1 shows an overview of the described cases.

**Table 1.** Overview of inpatient cases with infections following medical leech therapy.

	Case 1	Case 2	Case 3	Case 4	Case 5
Age	34 years	63 years	58 years	64 years	58 years
Sex	Male	Male	Male	Female	Female
Occupation	Office-based	Office-based	Office-based	None	Office-based
Pre-existing conditions	None	None	None	Diabetes mellitus, insulin-dependent	Polyarthrosis
Eaton/Littler stage before intervention	I	II	II	III	II
Hand dominance	Right	Right	Right	Right	Right
Affected hand	Right	Left	Right	Right	Right
Latency between leech therapy (LT) and symptoms	Redness, swelling, and pain 24 h after LT	Redness, swelling, and pain 24 h after LT	Redness and swelling 48 h after LT	Redness, swelling, pain, and a throbbing sensation 24 h after LT	Redness, swelling, and pain 48 h after LT
Intraoperative findings and surgical treatment	No pus, synovectomy	No pus, synovectomy	Pus	No pus, synovectomy of APL, EPB, EPL	Pus extending into TCJ, synovectomy
Inflammatory parameters at admission	CRP 0.5 mg/dL, normal leukocyte count	CRP 0.73 mg/dL, leukocyte count 11,300/µL	No pathological findings	CRP 1.5 mg/dL, normal leukocyte count	CRP 1.8 mg/dL, normal leukocyte count
Pathogen identified	No pathogen identified	Staph. epidermidis	A. hydrophila	P. aeruginosa	Staph. aureus
Antibiotic therapy	Cefuroxime IV × 7 days, amoxiclav PO × 7 days	Cefuroxime IV × 7 days, amoxiclav PO × 7 days	Ciprofloxacin IV × 5 days, no oral antibiotic therapy	Cefuroxime IV × 5 days, piperacillin/tazobactam IV × 9 days based on antibiogram, no oral antibiotic therapy	Flucloxacillin IV × 6 days, no oral antibiotic therapy
Number of surgical procedures	1	1	2	3	2
Postoperative management	Immobilization for 2 weeks (splint), hand therapy × 24	Immobilization for 2 weeks (splint), hand therapy × 12	Immobilization for 2 weeks (splint), hand therapy × 30	Hand therapy × 36	Hand therapy × 30
Length of hospital stay	7 days	7 days	5 days	14 days	7 days
Duration of work incapacity	4 weeks	3 weeks	9 weeks	–	–
Eaton/Littler stage after intervention	–	–	IV	IV	III
Result	Full range of motion in all planes	Full range of motion in all planes	Full range of motion in all planes	Full range of motion in all planes	Full range of motion in all planes

## Discussion

Medical leech therapy has been described as a treatment option for pain relief in patients with chronic pain conditions and osteoarthritis of the thumb carpometacarpal joint [6]. For example, the study by Michalsen et al. demonstrated pain relief and an improvement in hand function, including grip strength, following leech therapy in patients with rhizarthrosis [6]. Likewise, Hohmann et al. reported beneficial effects of leech therapy in patients with chronic low back pain [10].

Medical leech therapy is generally regarded by patients as a natural and risk-free treatment option. The aim is to achieve healing through complementary or alternative treatment approaches and to avoid surgical intervention. The literature, however, only provides limited data regarding the risks associated with medical leech therapy for rhizarthrosis. In particular, the risk of infection has received little attention.

Reported infection rates range from 2.4% to 36.2% [2]. Data on the severity of the clinical course and appropriate treatment strategies are scarce in the literature.

In our case series, the pathogen most commonly described as the causative agent of leech-associated infections was identified only in Case 3. In one of the cases, no pathogen could be identified despite the presence of an infection. It should also be considered whether the infections in the patients with S. epidermidis and S. aureus (Cases 2 and 5) resulted from contamination of the leech’s biting apparatus or inadequate skin cleansing prior to leech therapy.

In Cases 1, 2, and 4, a second-generation cephalosporin (cefuroxime) was selected for the treatment of the infection in addition to surgery. In Case 3, ciprofloxacin was chosen. This choice of antibiotic is consistent with established treatment recommendations for infections caused by Aeromonas hydrophila [5].

Furthermore, strategies to reduce the risk of infection associated with medical leech therapy warrant further discussion. As a possible approach, some authors advocate prophylactic antibiotic administration, such as ciprofloxacin, prior to medical leech therapy [4,5]. Pourrahimi et al. also reported that infections caused by resistant Aeromonas strains have been observed and emphasized that careful selection of antibiotic agents is an important factor in preventing further resistance [5]. Bykowski et al. reported ceftriaxone resistance in Aeromonas hydrophila in a case report. In addition, infections caused by ciprofloxacin-resistant strains have also been described in the literature [1,4]. One possible explanation discussed by the authors is the feeding of medicinal leeches with poultry that had previously been exposed to antibiotics during fattening [1]. As a potential solution, Pourrahimi et al. described combination antibiotic therapy in contrast to monotherapy [5].

The collection of microbiological samples from the rearing tank prior to the medical use of leeches has been recommended for testing antimicrobial susceptibility [1,5]. Based on the results of this prior testing, the choice of antibiotic could be adjusted accordingly.

Another approach described in the literature involves prophylactic antibiotic treatment of the leech population prior to use for medical purposes with ciprofloxacin or cefotaxime [5]. Prophylactic antibiotics could be administered when feeding the leeches sheep’s blood and additionally by adding antibiotics to the water in the rearing tank [5].

However, such an approach may undermine the intention of many patients to avoid conventional medical treatment. From the authors’ perspective, the routine use of antibiotic therapy solely to avoid established conservative and/or surgical treatment options for rhizarthrosis does not appear to be justified.

Since medical leech therapy has been shown to relieve pain according to the literature, but this effect is predominantly limited to a relatively short period of time [6,10], it should be carefully considered whether this treatment is truly appropriate.

The five cases presented here demonstrate that infection following medical leech therapy is a potential complication that can have serious clinical consequences. In all cases, at least one surgical intervention was required. In three cases, revision surgery in the form of a second-look procedure was required. All patients were treated on an inpatient basis and received intravenous antibiotic therapy tailored to the identified pathogen spectrum. The duration of antibiotic treatment was determined according to the local findings. All patients showed good hand function after completion of treatment. However, imaging revealed marked progression of rhizarthrosis in three cases, with advancement to a higher Eaton/Littler stage due to infection-related damage to the cartilage surfaces of the thumb carpometacarpal joint [3].

In summary, it can be concluded that infection following medical leech therapy for rhizarthrosis represents a serious complication. In many cases, these infections are caused by specific pathogens [1]. However, the present case series demonstrates that other pathogens may also be involved. Treatment of these infections generally consists of surgical intervention combined with systemic antibiotic therapy. Various approaches for the prevention of such infections have been proposed in the literature [5].

In the authors’ opinion, patients should receive comprehensive information regarding medical leech therapy, including its potential risk of infection, before treatment is initiated.

In our view, this risk is not sufficiently emphasized in the current literature on this treatment modality. The severity of the infections and their clinical course, together with the need for invasive treatment and long follow-up, absence from work, and the associated healthcare costs observed in the present case series, should draw attention to these potential complications.

Whether medical leech therapy is an appropriate treatment option for rhizarthrosis, considering its short-lived pain-relieving effect and lack of influence on disease progression, is not addressed in the present case series. Nevertheless, this treatment modality is not offered at our institution, as established conservative and surgical treatment options for rhizarthrosis generally achieve high patient satisfaction, substantial pain relief, and excellent thumb carpometacarpal joint function.

## Data Availability

The authors confirm that the data supporting the findings of this study are available within the article.
